# Amphibian and Reptile Road Mortality in Special Nature Reserve Obedska Bara, Serbia

**DOI:** 10.3390/ani12050561

**Published:** 2022-02-23

**Authors:** Marko Anđelković, Neda Bogdanović

**Affiliations:** 1 Department of Evolutionary Biology, Institute for Biological Research “Siniša Stanković”—National Institute of Republic of Serbia, University of Belgrade, Bulevar despota Stefana 142, 11060 Belgrade, Serbia; 2 Department of Animal Ecology and Zoogeography, Faculty of Biology, Institute of Zoology, University of Belgrade, Studentski trg 16, 11000 Belgrade, Serbia; neda.bogdanovic@bio.bg.ac.rs

**Keywords:** roadkill, herpetofauna, hotspots, vehicle collisions, road mitigation

## Abstract

**Simple Summary:**

Road networks allow for faster and easier transportation of people and goods, but unfortunately, they also have many negative effects on nature. For example, due to vehicle collisions, millions of animals are killed or injured every year. Usually, the effects of road mortality are not equally distributed in time and space. The main aim of this study was to examine patterns of amphibian and reptile road mortality on the road near the largest wetland in Serbia, on the edge of the Special Nature Reserve “Obedska bara”. During the 32 fieldwork days in 2018, we found more than 20,000 road-killed amphibians and reptiles, with amphibians accounting for 93% and reptiles 7% of the total fatalities. The numbers of killed amphibians and reptiles were not uniform between different habitat types and seasons. Therefore, an examination of the temporal (hot moments—periods when mortality rates are highest) and spatial patterns (hotspots—road sections with a higher concentration of vehicle collision) of roadkill is necessary for applying effective mitigation measures, which can reduce the negative impact of traffic.

**Abstract:**

Amphibians and reptiles are the most threatened vertebrates by traffic, especially near ponds and wetlands. The main aim of this study was to examine seasonal and spatial patterns of amphibian and reptile road mortality in Special Nature Reserve “Obedska bara” (Serbia). We chose a road section of 4.2 km near the largest wetland in Serbia, with two different habitat types (forest-pond and agricultural area-pond). During 2018 (32 fieldwork days) and 2019–2020 (three control fieldwork days), we found 20,457 and 2231 road-killed animals, respectively. We recorded nine amphibian and eight reptile species. Amphibians (93%) were more vulnerable to traffic than reptiles (7%). The number of killed amphibians and reptiles varied between the seasons. Generally, amphibian roadkill was most frequent during the summer, whereas reptile roadkill was most frequent in spring and at the end of the summer. Furthermore, different roadkill patterns were observed during the examined months. In addition, we found that habitat type significantly affects the vulnerability of animals towards the roads. For both amphibians and reptiles, there were significant aggregations of roadkill, and most hotspots were located at the forest pond habitat type where mitigation measures must be applied (construction of tunnels and fences).

## 1. Introduction

New roads are constantly being built for the needs of modern society. Road networks allow faster and easier transportation of people and goods, but unfortunately they also have many negative effects on nature [1,2,3,4]. Roads and traffic have: (1) direct effects which involve injury or mortality that occur during road construction [2] and due to collision with vehicles, and (2) indirect effects which include habitat loss and degradation [2], changes in microclimate conditions [5], as well as an increased level of noise [6], light [7], and chemical pollution [8]. These effects can result in a reduction in population size and density [9,10], reducing genetic diversity [11] and reproductive output [12], decreasing survival rates, and changing age and sex ratios [13,14], as well as animal movements (such as road avoidance or/and barrier effect) [1,2]. The negative impact is usually the greatest closer to the roads, but it can be observed in the road-effect zone which can cover a few hundred up to several thousand meters from the road itself [15]. Due to vehicle collisions, millions of animals are killed or injured every year (reviewed in [16]): invertebrates [17], amphibians [10,18,19], reptiles [18,20], birds [21] and mammals [1]. In addition to numerous animal deaths, results of collisions can cause significant material damage, as well as human injuries and death [22,23].

The number of studies related to killing of amphibians and reptiles on roads is increasing [19,24,25]. Roads and traffic, through habitat loss and fragmentation, greatly contribute to the endangerment of reptiles [26] and especially amphibians, as one of the most endangered vertebrate classes [27], and the group with the highest road mortality rate [28,29]. Both amphibians and reptiles are ectothermic animals, and they have different abilities (crossing speed) and behaviour (basking on the road) at different thermal conditions [30].

Amphibians have complex life cycles and often seasonally migrate because they require interconnected areas of land and freshwater for breeding, larval development, feeding, hydration, and hibernation [31]. Reptiles also migrate in search of optimal types of habitats for feeding, mating, basking, sheltering, oviposition, and hibernation [13].

Many factors, such as temperature, precipitation, fluctuations in water level, and photoperiod, can have a strong impact on amphibian and reptile activity and behaviour, which can be related to high roadkill rates [20,32]. The negative road effects depend on the geographical position of the road itself, surrounding habitats, the main road characteristics, the distance from the water surfaces, traffic volume, vehicle speed, and drivers’ behaviour [10]. Roads near wetlands are a cause of high amphibian and reptile mortality [28,30].

The effects of road mortality are not equally distributed in time and space [33]. Therefore, examination of the temporal (hot moments—periods when mortality rates are highest) and spatial patterns (hotspots—sections with a higher concentration of vehicle collision) of roadkill is necessary for applying effective mitigation measures [34]. Building fences, tunnels, and crossing structures can reduce animal mortality caused by vehicle collisions, but mitigation structures are expensive; thus, it is necessary to determine the roadkill hotspots and find the best places for the implementation of mitigation measures according to them [35,36,37].

The main aim of this study was to analyse roadkill data in order (i) to investigate which species were killed on the road and their roadkill rates, (ii) to examine seasonal and spatial patterns of amphibian and reptile road mortality, and (iii) to test whether roadkills occurred randomly or they were spatially clustered in hotspots.

## 2. Materials and Methods

### 2.1. Study Area

This study was conducted in the Special Nature Reserve “Obedska bara”, which is in the northern part of Serbia (50 km from the capital city—Belgrade). The first administrative protective measures were introduced in 1874 [38], making the Obedska bara one of the oldest protected areas (after Yellowstone) in the world. The Special Nature Reserve is the largest wetland in Serbia. Its status has been verified by the Ramsar Convention since 1977. Obedska bara is characterised by a moderate continental climate with most precipitation during the warm half of the year (especially during May and June). The main natural value of the Special Nature Reserve is the ecosystem integrity with a remarkably rich ecosystem and species biodiversity, which includes rare and endangered species of national and international significance. Eleven species of amphibians (*Pelophylax ridibundus*, *P. lessonae*, *P. kl. esculentus*, *Pelobates fuscus*, *Hyla arborea*, *Bombina bombina*, *Rana dalmatina*, *Bufo bufo*, *Triturus dobrogicus*, *Bufotes viridis*, *Lissotriton vulgaris*) and eight species of reptiles (*Natrix natrix*, *Natrix tessellata*, *Zamenis longissimus*, *Coronella austriaca*, *Vipera berus*, *Emys orbicularis*, *Lacerta viridis*, *Lacerta agilis*, *Anguis fragilis*) live in the Special Nature Reserve “Obedska bara”.

**Figure 1:**
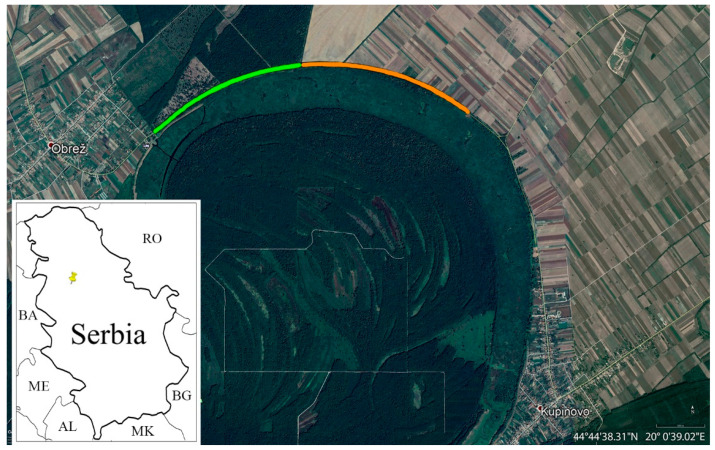
Position of the Special Nature Reserve “Obedska bara” on the map of Serbia and aerial map from Google Earth of the analysed road section in Special Nature Reserve “Obedska bara”, between Obrež and Kupinovo. The first section with forest habitat is green, whereas the second section with agricultural land is orange.

We were monitoring the traffic effects on the amphibian and reptile populations on the local road Obrež-Kupinovo (Figure 1). This is a two-lane paved road about 5 m wide. We chose a road section of 4.2 km located on the edge of Special Nature Reserve “Obedska bara”, which we divided into two sections of the same length. The first section is characterised by forest habitat, whereas the second is characterised by agricultural land. The examined road is located between these habitats and the pond (Figure 1).

### 2.2. Data Collection

Data collection was performed during 32 field days in 2018 (from March to December). We divided the sampling period into three seasons: spring (12 surveys from 30 March to 20 June), summer (12 surveys from 28 June to 19 September), and autumn (8 surveys from 29 September to 30 November). Data were also collected during the three control field days in 2019 (March and October) and 2020 (April), to compare the patterns recorded during 2018. We (M.A. and N.B.) surveyed the road for animal roadkill on foot because it has been shown that this method is more efficient than others [37,39], with a monitoring frequency of 3–10 days during 2018. The research was conducted during the day from 8 to 15 h. On several occasions we checked the road section twice (in both directions) to determine the success rate of roadkill detection, and it was greater than 95 per cent. We removed dead animals from the road to avoid double counting. Road-killed animals were identified, and for each specimen we recorded the date and GPS coordinates (using the Android application GPS Essentials).

### 2.3. Data Analysis

To determine whether there were significant differences in the number of dead animals per taxon (reptiles, amphibians), between seasons (spring, summer, autumn) or between habitat types (pond-forest and pond-agricultural area), we performed chi-squared tests.

To determine the scales on which roadkill were significantly aggregated in space, we used a modified Ripley’s K statistic [40] in Siriema v2.0 software (www.ufrgs.br/biociencias/siriema (accessed on 16 July 2018)), with the following parameters: initial radius of 50 m, increments of 50 m for each step [40], and a 1000 Monte Carlo simulations of a random roadkill distribution for each scale. After the simulations, values above the confidence limits (95%) indicate scales with significant aggregations [41].

To identify the location of hotspots, we performed a HotSpot Identification analysis [42] using the Siriema software. For this analysis, we divided the road into the same length segments (50 m) and a circle of a 100 m radius was centered at the midpoint of the first segment, summing the values for all roadkill events inside the circle area. This sum was multiplied by a correction factor that considers the length of the road analysed inside the circle in this position. This procedure was repeated for all segments, resulting in a roadkill aggregation intensity value for each segment of a road [43]. We considered that hotspots were all segments with a roadkill intensity value higher than the upper confidence limit of 95% after 1000 simulations of random distribution [43]. For hotspot analysis of amphibians and reptiles (when species are grouped) we used different weight for species based on protection levels in Serbia [44] (value 1 in Z column for *Anguis fragilis*, *Lacerta viridis* and *Lacerta agilis* which are not protected, value 2 in Z column for *Pelophylax esculentus* complex (*Pelophylax ridibundus*, *P. lessonae*, *P. kl. Esculentus*) which is protected, value 3 for the remaining species which are strictly protected), and when the analysis was performed for each species separately, we set the same value (1) on column Z [43]. Because the road section is linear and the preliminary analysis showed pretty similar results, we used a linear, as opposed to a two-dimensional, K-function and hotspot analysis. Before all analyses in Siriema, we used the “Fit events” function which relocates the roadkill events with small errors on coordinates to the shortest distance possible to the road track [43].

In order to estimate road mortality rate (roadkill per day and roadkill rate per kilometre), we used the function “Mortality rate estimate” with the following parameters: road length (4.2 km), the total number of roadkill for each taxon, searchers’ efficiency (*p* = 0.9, [42]), carcass removal characteristic time (TR(day) = 0.96 for amphibians and 2.45 for reptiles [37]), number of surveys (32) and sampling interval (TS(day), we used mean value 6.9).

## 3. Results

### 3.1. Number of Amphibian and Reptile Roadkills

During the fieldwork in 2018, we found 20,457 road-killed amphibians and reptiles, including nine amphibian and eight reptile species (Table 1a). Amphibians (93%) were more vulnerable to traffic than reptiles (7%). Specimens of the *Pelophylax esculentus* complex are most frequently killed amphibians, followed by *Pelobates fuscus*, whereas specimens of *Natrix natrix* were most frequently killed among reptiles, followed by *Emys orbicularis* (Table 1a). For *Lissotriton vulgaris* and *Coronella austriaca*, we found only one killed specimen. Twenty-one frogs were impossible to identify at the species level, and these specimens were not used in hotspot analyses.

During control fieldwork we recorded 2231 road-killed animals and observed a similar road-killed pattern (amphibians 76%; Yates’ chi-square = 609.393, *p* = 0.001) (Table 1b). The same amphibian species composition and a smaller number of reptile species were recorded in this sample. Additionally, specimens of *P. esculentus* complex and *Natrix natrix* are most frequently road-killed (Table 1b).

### 3.2. Seasonal Patterns of Roadkill

**Figure 2:**
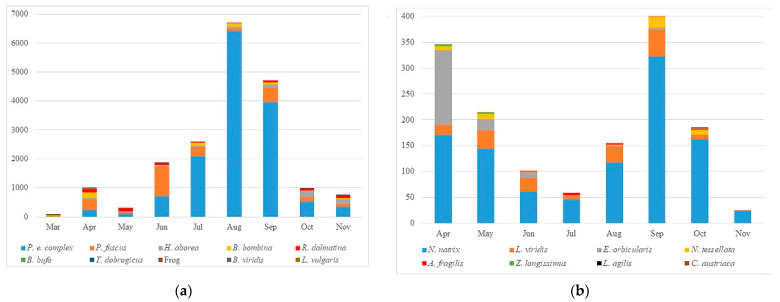
The number of road-killed individuals per month: (**a**) road-killed amphibians; (**b**) road-killed reptiles.

The number of road-killed amphibians and reptiles (corrected for the number of days per season) is not uniform during the seasons (Yates’ chi-square = 1048.322, *p* < 0.001; Yates’ chi-square = 8.179, *p* = 0.017; respectively). Amphibian roadkill were most frequent during the summer, with a peak in August (*n* = 6688), followed by September (*n* = 4710), and the main contributors were species from the *P. esculentus* complex (Figure 2a). Other species do not have the same roadkill patterns during the months (Figure 2a). *Bombina bombina* had peaks in April and August (July–September); *Bufo bufo* and *Triturus dobrogicus* had peaks in April and November. The number of killed individuals of *Hyla arborea* has been increasing since the beginning of the season and it reached a peak in October. Road mortality of *P. fuscus* varies greatly over the months, with the biggest peak in June. *Rana dalmatina* has a similar road mortality rate during the investigated period, except during June and July when the number of road-killed individuals was significantly lower. *Bufotes viridis* was found only in March and Jun, whereas *L. vulgaris* was found only in November.

Road mortality of reptiles was double-peaked: the first peak occurred at the beginning of the spring in April (*n* = 346) and the second one occurred at the end of the summer in September (*n* = 404) (Figure 2b). The main contributors were *N. natrix* and *E. orbicularis* which had a significant contribution to the first peak. Other species did not show the same pattern (Figure 2b). Road-killed specimens of *L. agilis* were found only in September and October. Similarly, we found only one specimen of *C. austriaca* in September. *L. viridis* and *Natrix tessellata* had two peaks in May and September, whereas *Zamenis longissimus* had peaks in April–May and October. *Anguis fragilis* had a similar roadkill rate from June to October.

Control fieldworks were not covered all seasons; therefore, seasonal patterns were not analysed for these data.

### 3.3. Spatial Patterns of Roadkill

**Figure 3:**
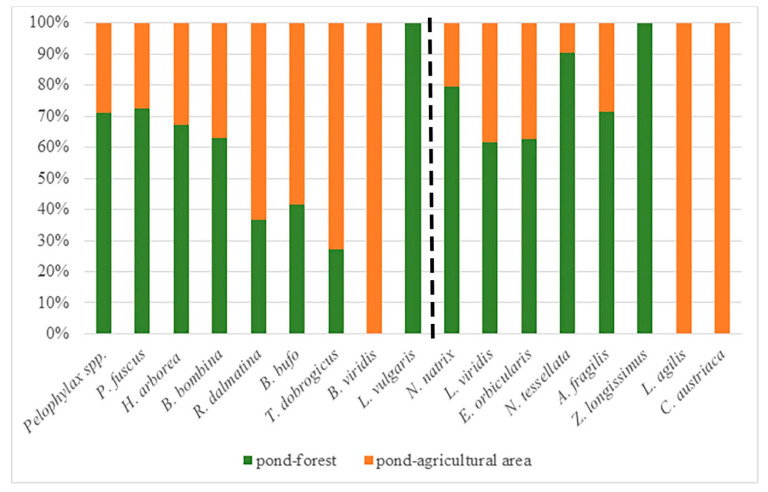
The proportion of road-killed amphibians and reptiles on the road sections with different habitat types (pond-forest and pond-agricultural area).

The total number of road-killed amphibians and reptiles was higher in the first section with a forest habitat than in the second road section with an agricultural area (Yates’ chi-square = 2929.41, *p* = 0.001; Yates’ chi-square = 386.632, *p* = 0.001; respectively). When we analysed species separately, we noticed a similar pattern for most species except for *R. dalmatina*, *T. dobrogicus* and *B. viridis* (Figure 3). The obtained results also showed that there is a difference in species composition: *L. vulgaris* and *Z. longissimus* were found only in the forest habitat, whereas the species *B. viridis*, *C. austriaca* and *L. agilis* were found only in the road section with an agricultural area (Figure 3).

**Figure 4:**
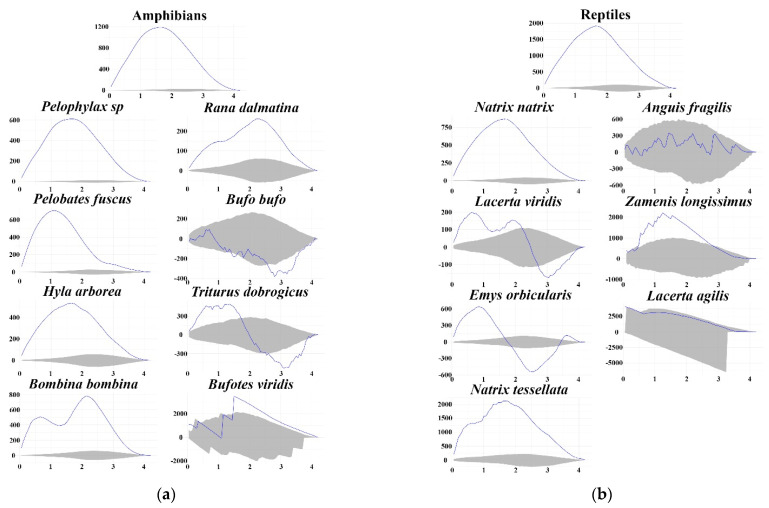
Results of Ripley’s K analysis presented as roadkill aggregations (*y*-axis) according to the radius (*x*-axis in km) for (**a**) amphibians and (**b**) reptiles. Blue line represents aggregation intensity. 95% confidence limits are represented by grey area. Significant aggregations of road mortality events occur if the blue line exceeds the upper confidence limit. Values under confidence limits indicate scales with significant dispersion. The figure was made using the Siriema plots application [45].

The results of Ripley’s K analyses showed that for both amphibians and reptiles, there was a significant aggregation of roadkill, but when the species were analysed separately, there were two exceptions (*B. bufo* and *A. fragilis*) (Figure 4). The scale of the roadkill aggregations on the road differed between species, showing that *L. agilis* has the smallest range (10–70 m), whereas species from *P. esculentus* complex, *P. fuscus*, *H. arborea*, *B. bombina*, *R. dalmatina*, *N. natrix* and *N. tessellata* have shown a much larger range (almost the entire analysed road distance).

**Figure 5:**
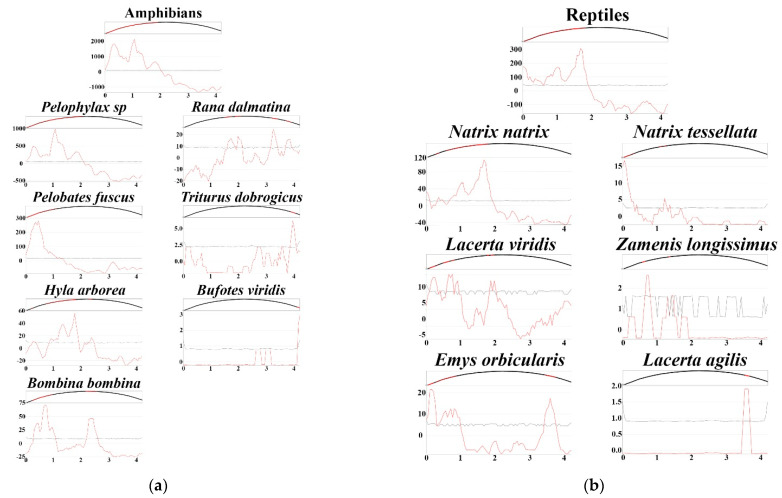
Spatial distribution of hotspots for (**a**) amphibians and (**b**) reptiles. *X*-axis represents road length (km), whereas *y*-axis represents roadkill intensity. Grey line indicate 95% confidence interval, and red line represents the spatial aggregations of the roadkill data. Values that exceeded the confidence interval indicate the hotspots. Above each graph the analysed road section is given with hotspots which are marked with red colour. The figure was made using the Siriema plots application [45].

Based on the results from the Ripley’s K analysis we performed HotSpot Identification analyses which indicated that hotspots for amphibians and reptiles (species grouped within higher taxon) were concentrated almost completely in the first section (Figure 5). A similar result was found for *P. esculentus* complex, *N. natrix*, *P. fuscus*, *L. viridis*, *N. tessellata* and *Z. longissimus* which had roadkill aggregations only on the first road section (Figure 5). *H. arborea*, *B. bombina*, *R. dalmatina* and *E. orbicularis* had roadkill aggregations on both analysed road sections, whereas *T. dobrogicus*, *B. viridis* and *L. agilis* had hotspots localised only on the second road section (Figure 5).

Data obtained from the control fieldwork confirmed that amphibians and reptiles are more vulnerable on the first section (Yates’ chi-square = 1126.235, *p* = 0.001, Yates’ chi-square = 526.017, *p* = 0.001, respectively). HotSpot Identification analyses also confirmed this result (Figure S1).

### 3.4. Mortality Rate

We estimated a mortality rate of 160.1 roadkill/day/km for amphibians and 5.3 roadkill/day/km for reptiles on the analysed road section (Table 1a). Based on data from the control fieldwork, similar values for the mortality rate were observed in amphibians, but significantly higher in reptiles (Table 1b).

## 4. Discussion

### 4.1. Number of Amphibian and Reptile Roadkills

Roads crossing results in a large number of road-killed animals. For example, in the USA approximately one million vertebrates are killed each day, and Ehmann and Cogger [18] noted that five million reptiles and amphibians are killed annually on Australian highways. Because in Serbia there were no systematic studies related to road mortality, we were investigating the effects of traffic on amphibians and reptiles at the Special Nature Reserve Obedska bara, where more than 20 years ago, Pantelić [46] carried out five-day monitoring and pointed out the problem of killing amphibians and reptiles on the road. We found more than 20,000 road-killed amphibians and reptiles, with amphibians accounting for 93% and reptiles 7% of the total fatalities. The higher mortality of amphibians compared with reptiles was recorded by other authors as well [30,47]. The number of road-killed individuals per species varies widely (Table 1a,b), which may be due to several factors. We found that specimens from *P. esculentus* complex, which are mostly aquatic frogs, are the main contributors of the total roadkill. Contrary to our findings, few studies revealed that more active and terrestrial amphibians are more likely to be killed on the roads [3]. The reason for this result may be the local population abundance [48]. A large number of road-killed green frogs may be unexpected because we predominantly found them near water, but Günther [49] noted that they regularly explore terrestrial habitats and have overland migrations. *Bombina bombina* which is also an aquatic species that lives in shallow waters, was often found killed on the road. This observation is partly because individuals of *B. bombina* use drainage channels and temporary ponds (filled with rainwater) near the road for their dispersion.

Among terrestrial species, we unexpectedly found many killed individuals of *P. fuscus*. In his study, Pantelić [46] also noted that the individuals of *P. fuscus* accounted for 25% of the total road-killed animals. *Pelobates fuscus*, as well as *B. bufo* and *B. viridis*, are predominantly active during the night [10] when there is probably lower traffic intensity. However, it has been shown that even small traffic intensity can cause a high level of road mortality [50]. In our case, *H. arborea* takes third place among the amphibian roadkill, which is contrary to the results obtained by Elzanowski and collaborators [29], who noted that *H. arborea* is rare in the roadkill records on the European roads. Individuals of *R. dalmatina* were also commonly found, which may not be expected because of its good jumping ability, a short breeding period in the early spring, and a higher level of terrestrial lifestyle. However, Hartel and collaborators [51] found that *R. dalmatina* spends more time on the road than it needs to cross it. Road mortality rates of *B. bufo*, *T. dobrogicus*, *B. viridis* and *L. vulgaris* were significantly lower than expected. *Bufo bufo* is the most killed species on European roads [29], and a lower road mortality rate in our case may be due to lower abundance, nocturnal activity or because we only partially captured its migration due to the large range between two surveys. *Triturus dobrogicus* and *L. vulgaris* are recorded in small numbers, perhaps because they have a small and soft body; therefore, they are probably more difficult to spot and disappear more quickly from the road [52]. A small number of road-killed individuals of *B. viridis* may be due to their small population, nocturnal activity or because they are more associated with a human settlement that has not been analysed in this study [53].

*Natrix natrix* was the most killed reptile species, similar to the results obtained in the research of Heigl and collaborators [54]. The reasons for that can be their abundance, a large home range [55], as well as the search for green frogs, which are very numerous in the road zone. The road mortality rate of *N. tessellata* was lower since it is a piscivorous species that is predominantly located near water surfaces [56]. Similar results have been obtained for two species from the genus *Nerodia* [57]. Specimens of *Z. longissimus* and especially *C. austriaca* were found in smaller numbers since these species have lower population densities compared with water snakes [58]. One of the reasons for snake road mortality is their use of roads for thermoregulation [59]. *Emys orbicularis* and *L. viridis* occupy second place among the road-killed reptiles. We often saw individuals of *L. viridis* in the road zone as they were eating, defending their territory, running from one side to the other, and in these activities many individuals were overstretched, and some lost their tail. Turtles are slow-moving animals, especially young individuals who need a lot of time to cross the road [60]. As noted for amphibians [61], some turtles and snakes become immobile in response to an approaching vehicle [62,63]. Turtles have delayed sexual maturity and low fecundity [64], which is why vehicle collisions may have a strong impact on turtle populations [60]. Individuals of *A. fragilis* had a low road mortality rate, perhaps because of its secretive lifestyle, although Wells and colleagues [65] have noted that *A. fragilis* are common in roadside habitats. Individuals of *L. agilis* were found in low numbers, since this is not their optimal habitat.

### 4.2. Seasonal Patterns

The greatest mortality of amphibians is expected in the spring and autumn when the biggest migrations occur between the hibernation site and the breeding site [66]. This pattern was found only for *R. dalmatina*, *T. dobrogicus* and *B. bufo*, but not for other species and when amphibian species are grouped. For individuals from *P. esculentus* complex and *B. bombina* we found that roadkill rates are greater during the summer, for *P. fuscus* and *B. viridis*, the mortality rate decreases from spring to autumn, whereas on the other side, the mortality rate of *H. arborea* increases from spring to autumn. These different patterns can reflect species-specific needs and characteristics of life history: different mating periods, time spent in the water, different tolerances on weather conditions and different dispersion periods of individuals [67]. Since the temperature of the amphibians depends on the surrounding environment, they are exposed to desiccation, and their migration is limited to periods with optimum temperature and humidity. It is known that temperature and precipitation have a strong effect on anuran roadkill [42]. In our case, there was a lot of precipitation during the summer months, and as a result of frequent rain the dents along the road (which can be attractive to amphibians) were filled with rainwater, thus they could result in many road-killed individuals.

In reptiles (in most species and when species are grouped) we noticed higher roadkill rates during spring and/or autumn months. The exception is *A. fragilis*, because its specimens were predominantly killed during the summer months. During the year, reptiles have different activities that can be related to their presence on the roads. In the spring, they leave the hibernacula’s and start looking for a mating partner, whereas during the summer they are less active, focusing their activity on searching for food and suitable places for basking [30,68] and laying eggs [69]. During the autumn, their activity increases when they intensively feed (preparation for hibernation) and search for the appropriate place for hibernation. In addition, changes in weather conditions can be a trigger for their movements, resulting in an increase in mortality [12].

We would like to note that the mentioned seasonal differences were obtained on the basis of one year of research. However, as mentioned in the introduction, many factors can affect the activity of amphibians and reptiles [10,20,32]; thus, examining the temporal pattern of amphibian and reptile road mortality requires long-term monitoring of the roadkill rate, as well as other factors (temperature, precipitation, traffic intensity, population density).

### 4.3. Spatial Patterns

As we expected for most species, hotspots were located on the road section with the forest habitat. This type of habitat probably offers a greater possibility of hiding from predators, more favourable temperature conditions, more appropriate sites for egg incubation, and increased availability of prey in relation to the agricultural land where people often use different chemicals to suppress pests (insects and rodents), which are amphibians’ and reptiles’ prey.

Shepard and associates [59] have shown that road segments that pass through a variety of habitats (a large number of shelters, a larger prey spectrum, etc.) have a higher reptile roadkill rate. Spatial aggregation patterns may also be influenced by the philopatry [70].

### 4.4. Mortality Rate

The number of road-killed animals and their mortality rates are probably much greater because many animals who are hit are never detected on the road [37]. For example, Santos and colleagues [71] suggested that the real number of roadkill is at least 2–10 times greater than estimated. High road mortality may occur on just a few rainy days, but these events may be difficult to detect due to the high range between surveys, mechanical destruction by vehicles [10,50] or killed animals can be eaten by scavengers [20,72]. In relation to the previous statement, we have also observed birds eating or carrying roa-killed animals during our research. Carcass persistence on road is different, and it depends on weather conditions and traffic frequency [39,72]. DeGregorio and colleagues [73] found that 70% of road-killed snakes were removed by scavengers within 16 h, whereas Antworth et al. [74] documented that 97% of snake carcasses were removed within 36 h, maybe because snakes are more visible to birds than other reptiles. Previous studies have also shown that the average persistence time of “small carcasses” was 2 days, with persistence probability dropped after one day [37,39,71]. In our case, “small carcasses” are a heterogeneous group, and specimens of reported species have different sizes, shapes and structures, so it is likely that their presence on the road is different. For example, Hels and Buchwald [10] noted that by checking the road on foot once in 24 h you can find only 7% (*Triturus vulgaris* and *T. cristatus*) to 67% (*P. fuscus*) of the total number of road-killed specimens.

The mortality rate of amphibians and reptiles are difficult to compare due to the differences between study areas, road types, traffic frequency, species composition and different methodologies (different tracking periods and route inspection) [75].

Previous studies have reported lower roadkill rate (roadkill/km/year) for amphibians [19,30,42] and reptiles [30,40,50,76].

### 4.5. Impact of Amphibian and Reptile Road Mor Tality

The negative impact of roads on the amphibian and reptile populations is often underestimated [77], but they are an important part of natural ecosystems. For example, amphibians and reptiles are an important food source for higher trophic levels. Frogs, as well as bats and birds, play an important role in controlling the number of mosquitoes (and other pest insects). Snakes play an important role in the control of rodents which can cause serious damage to agricultural crops and are often transmitters of infectious diseases.

It is known that road mortality is associated with declines in amphibian and reptile populations that are directly exposed to anthropogenic influence, but these declines are recorded for populations which are not directly exposed to anthropogenic effects [9,30]. Anthropogenic activities that are mostly responsible for this trend of threatening amphibians and reptiles, as well as other taxa, are the destruction and fragmentation of habitats (deforestation, drainage of wetlands, creation of new agricultural areas, urbanization), various forms of environmental pollution (accumulated toxic substances can be transferred to higher trophic levels), and increased exploitation of natural resources. All mentioned activities are strongly related to the expansion of the road networks. Threatening amphibian and reptile populations can cause cascading processes and may result in a reduction in biodiversity, modified species composition, as well as spreading of allochthonous species.

Based on the rulebook on a compensation price list [78], the damage for the recorded road-killed amphibians and reptiles during 2018 amounts to over EUR 2 million, or much more if the estimated number of killed animals is taken into account.

To reduce the negative impacts of roads and traffic, appropriate protective measures need to be applied, which require a wide range of research [79]: basic roadkill surveys, analyses of animal movements and their home ranges; continuous monitoring of population parameters (size or density, survival rates, sex ratios and reproductive output). Based on the obtained results, it is possible to set the appropriate methods to reduce and prevent amphibian road mortality and to enhance connectivity between fragmented habitats (road signs, temporary fencing, wildlife crossing structures, road closures) [3,80]. The application of appropriate mitigation measures is especially needed for countries where there is no such practice, as is the case with Serbia and some neighbouring countries.

## 5. Conclusions

The extremely high number of amphibian and reptile roadkill found in the protected area requires the urgent application of appropriate protection measures in places marked as hotspots. This study indicates the importance of monitoring the traffic impact on animal populations near existing roads and conducting research before the construction of new roads. Special attention should be dedicated to the roads sections that pass near wet habitats, where many animals can be killed, as in our case. Further research on this topic is urgently needed because otherwise the road mortality of amphibians and reptiles may cause the destruction of entire ecosystems.

## Figures and Tables

**Table 1:** Number of roadkill for each species and higher taxonomic groups—N, per cent of total road-killed animals—N%, per cent of the road-killed group (amphibians or reptiles)—Ngroup, amount of roadkill per day and km, corrected for detectability biases. (searchers’ efficiency *p* = 0.9, carcass removal characteristic time TR = 0.96 for amphibians and 2.45 days for reptiles, and sampling interval TS = 6.9 days). (**a**) data from fieldwork during 2018 (32 fieldwork days); (**b**) data from control fieldwork (3 fieldwork days during 2019 and 2020).

(**a**)					
**Taxon**	**N**	**N%**	**Ngroup**	**N/Day**	**N/Day/km**
Amphibians	18,967	92.716	100	672.5	160.13
*Pelophylax* spp.	14,463	70.700	76.253	512.8	122.1
*Pelobates fuscus*	2591	12.666	13.661	91.87	21.87
*Hyla arborea*	646	3.158	3.406	22.91	5.45
*Bombina bombina*	587	2.869	3.095	20.81	4.96
*Rana dalmatina*	576	2.816	3.037	20.42	4.86
*Bufo bufo*	41	0.200	0.216	1.45	0.35
*Triturus dobrogicus*	37	0.181	0.195	1.31	0.31
Unidentified anurans	21	0.103	0.111	0.74	0.18
*Bufotes viridis*	4	0.020	0.021	0.14	0.03
*Lissotriton vulgaris*	1	0.005	0.005	0.04	0.01
Reptiles	1490	7.284	100	22.29	5.31
*Natrix natrix*	1048	5.123	70.336	15.67	3.73
*Lacerta viridis*	183	0.895	12.282	2.74	0.65
*Emys orbicularis*	182	0.890	12.215	2.72	0.65
*Natrix tessellata*	52	0.254	3.490	0.78	0.19
*Anguis fragilis*	14	0.068	0.940	0.21	0.05
*Zamenis longissimus*	8	0.039	0.537	0.12	0.03
*Lacerta agilis*	2	0.010	0.134	0.03	0.01
*Coronella austriaca*	1	0.005	0.067	0.01	0.01
(**b**)					
Amphibians	1699	76.15	100.00	655.48	156.07
*Pelophylax* spp.	1313	58.85	77.28	506.56	120.61
*Pelobates fuscus*	62	2.78	3.65	23.92	5.70
*Hyla arborea*	25	1.12	1.47	9.65	2.30
*Bombina bombina*	33	1.48	1.94	12.73	3.03
*Rana dalmatina*	249	11.16	14.66	96.06	22.87
*Bufo bufo*	1	0.04	0.06	0.39	0.09
*Triturus dobrogicus*	1	0.04	0.06	0.39	0.09
*Bufotes viridis*	14	0.63	0.82	5.40	1.29
Unidentified anurans	0	0.00	0.00	0.00	0.00
*Lissotriton vulgaris*	1	0.04	0.06	0.39	0.09
Reptiles	532	23.85	100.00	80.88	19.26
*Natrix natrix*	524	23.49	98.50	79.21	18.86
*Lacerta viridis*	4	0.18	0.75	0.60	0.14
*Emys orbicularis*	1	0.04	0.19	0.15	0.04
*Natrix tessellata*	3	0.13	0.56	0.45	0.11
*Anguis fragilis*	0	0.00	0.00	0.00	0.00
*Zamenis longissimus*	0	0.00	0.00	0.00	0.00
*Lacerta agilis*	0	0.00	0.00	0.00	0.00
*Coronella austriaca*	0	0.00	0.00	0.00	0.00

## Data Availability

The data presented in this study are available on request from the corresponding author. The data are not publicly available because the authors will use them in future research.

## Supplementary Materials

The following supporting information can be downloaded at: https://www.mdpi.com/article/10.3390/ani12050561/s1, Figure S1: Spatial distribution of hotspots for amphibians and reptiles based on control fieldwork. *X*-axis represents road length (km), whereas *y*-axis represents roadkill intensity. The gray line indicates a 95% confidence interval, and the red line represents the spatial aggregations of the roadkill data. Values that exceeded the upper confidence limit indicate the hotspots. Above each graph, the analysed road section is given with hotspots which are marked with red colour. The figure was made using the Siriema plots application [45].
