# Supplementary figures and images for: Amphibian and Reptile Road Mortality in Special Nature Reserve Obedska Bara, Serbia
Source: Animals (Basel). 2022 Feb 23;12(5):561. doi: 10.3390/ani12050561 (PMC8908848; doi:10.3390/ani12050561)

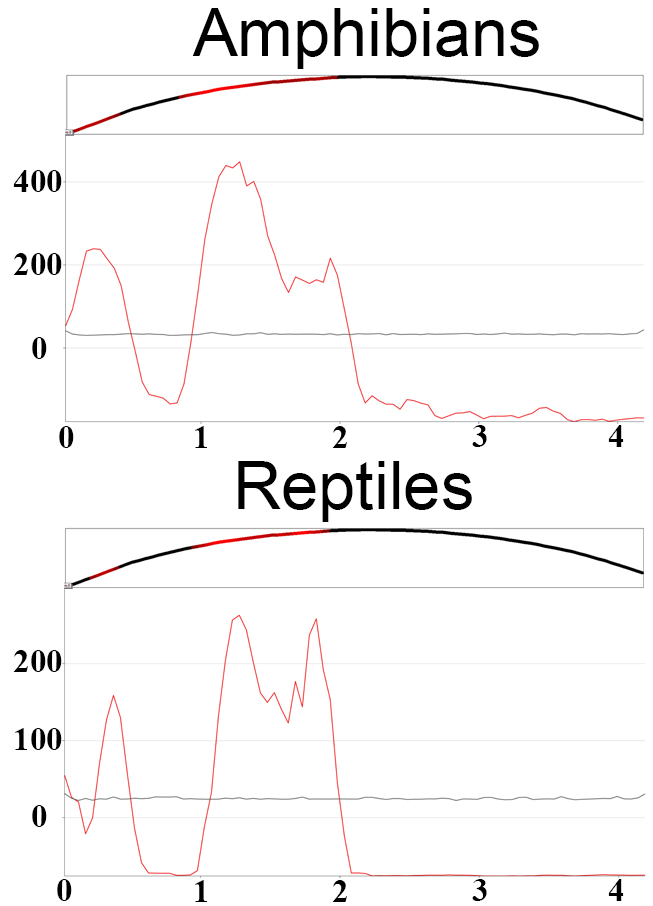

Supplement: Supplementary file 1 [file animals-12-00561-s001.zip › animals-1578359-Figure S1.jpg]
